# Immunity, Inflammation and Disease – Contributing to Quality Scientific Publishing

**DOI:** 10.1002/iid3.2

**Published:** 2013-10-30

**Authors:** Marc Veldhoen


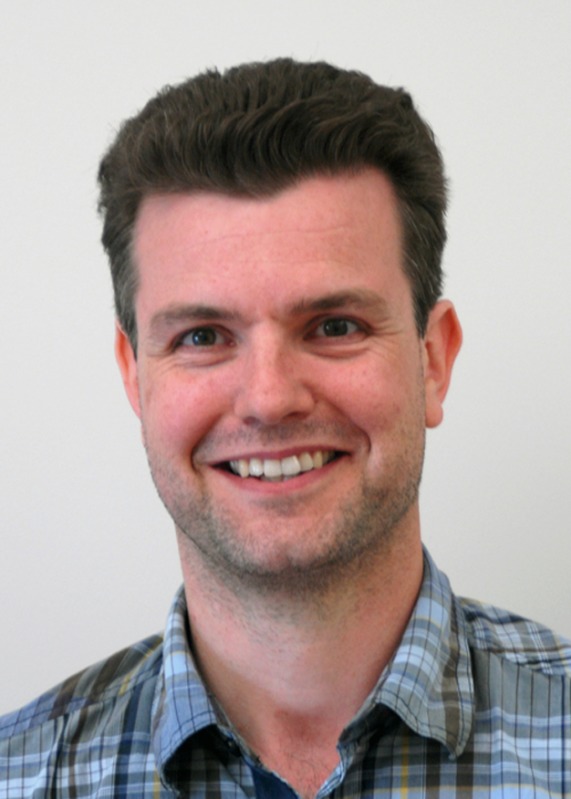


We are in the midst of a sea change in the process of publishing scientific findings and making them directly accessible to a wide audience. It is a huge waste of research funds and time if public funded research is not published, read and acted upon. We, as researchers, give careful thought to when and where to publish our results and interpretations. We take into consideration where our results will be seen by our peers, and thus potentially be cited, and whether we wish to reach a broad or more specialised audience. Funding bodies and governments are increasingly making it a requirement that the work they fund can be accessed and read by the widest possible audience. Unrestricted access to the published outputs of research is fast becoming the expected norm.

Many scientists are broadly in agreement with the idea of open access. But, and most importantly, our funding and careers critically depend on where our results get published. We need to maximise the impact and citations of our scientific contributions. The funding bodies demand that the output of their funded research is published in high-quality and peer-reviewed journals.

After the investment of considerable time and resources, we have the expectation that, often after a lengthy and sometimes frustrating process, the outcome of our carefully designed experiments will ultimately be accepted for publication. In addition, we envisage that it will have some influence on our colleagues' work and thinking, thereby advancing scientific progress. Picking the right journal has never been easy, and the appearance on the scene of open access journals with less upright reputations (1), together with the pressure from funders to make our studies open access, has not made this task simpler.

Despite all the work authors put into ensure good quality data and meaningful presentation and discussion, the contribution of editors and independent reviewers remains critical to ensure quality and methodological rigour. The fundamental scientific method of questioning results and discussing their interpretation ensures quality, improves impact and should facilitate progress. Although often criticised and with some inherent problems, this process of anonymous reviewing is the best we have. However, many manuscripts will not be accepted even after all this effort on the part of the authors and expert reviewers. This seems a waste of the time and effort of all involved. This is especially the case when reasons for rejection are as yet limited mechanistic insight or an insufficiently fashionable subject, or where precedence is given to novel studies over those containing solid experiments or clinical studies which strengthen previously published work. The original, independently made, observations themselves are certainly worthy of publication and discussion to further undertakings of others and to strengthen or undermine current theories.

Open access can put a strain on the system of peer review and scientific quality if not well managed. One might think, for instance, that it would be in a journal's interest to accept as many papers as possible, generating a stream of revenue. This would be a misconception; reputation and impact, key indicators of quality and thus success, would be lost. The new journals do not initially have the established indicator of quality, the somewhat controversial impact factor. Therefore, the model of open access publishing needs to be built upon quality, trust and transparency. Our journal depends critically on support from, and collaboration with, authoritative societies and other journals within its targeted readership, as well as on internationally renowned expert reviewers and editorial board members.

It is my hope and goal with *Immunity, Inflammation and Disease* to make a contribution towards quality open access scientific publishing, to stimulate debate and to make better use of the work of external reviewers. The journal is part of the respected Wiley Blackwell publishing house, making full use of its experience with quality peer reviewed scientific publishing. All research articles published in Wiley Open Access journals are immediately freely available to read, download and share. Importantly, *Immunity, Inflammation and Disease* is published in collaboration with the European Federation of Immunological Societies (EFIS), and the British Society for Immunology (BSI). It will give rapid consideration to manuscripts in all areas of clinical and basic immunology research for the attention of a broad readership in basic and applied immunology. We welcome original work that enhances understanding in the field of immunology, including methods papers, editorials, discussions and commentaries. Importantly, original research manuscripts need to report well-conducted experiments with conclusions that are supported by the data presented. In addition, *Immunity, Inflammation and Disease* will consider for publication both papers submitted directly to the journal and those referred from a select group of prestigious journals published by Wiley Blackwell. List available here.

All submitted manuscripts will undergo external peer review, or in case of referral are submitted with the original reviewer's comments. The latter enables us to make full use of the efforts already made by external reviewers and to make efficient use of their work and time as well as the authors' contribution, in energy, time and funds, to address the initial concerns. As an open access online journal there are no space constraints or demands, and short papers focussing on an initial novel observation or principle are highly encouraged. In addition, room for additional but focussed discussion to develop ideas further and to inspire others will be permitted. It is important that the data shown are correct with the methods used and available at the time, but the interpretation is allowed to be open for scientific discussion and debate. However, the absence of restraints on space does not imply that numerous supplementary figures should be submitted. Those included should substantiate primary figures (such as additional controls and video streams), should be directly related to a primary figure and should not outnumber the quantity of primary figures.

The process of anonymous external review will be conducted within two weeks and an editorial decision will be made within four to five weeks of submission, providing a rapid turnaround time. Importantly, additional experiments requested by external reviewers should be able to be conducted within a time frame of three months and need to be solely designed to strengthen or refute a fundamental claim made in the manuscript. Upon resubmission, the manuscript will be either accepted or rejected. Authors and external reviewers do not need to agree, but fundamental disagreements need to be highlighted in the discussion by the authors before final acceptance of the manuscript. This allows for the reader to have a better informed view of the data and its interpretation, and possibly to contribute to the discussion themselves with new data or arguments, and it enables the discussion or points of dispute to be cited in future publications. Transparency is very important and the publisher and I will regularly review and publish rejection and acceptance rates, times to online publication and other relevant information on the website.

My aims are to shape *Immunity, Inflammation and Disease* into a high quality journal publishing new and original data, to allow for novel data to be a bit rough around the edges, and to encourage strongly the preservation of the original scientific method of open discussion and frank exchange of thoughts and ideas.

